# Factors Related to Biomedical Research Productivity in Asian Countries

**DOI:** 10.2188/jea.11.199

**Published:** 2007-11-30

**Authors:** Mahbubur Rahman, Tsuguya Fukui

**Keywords:** biomedical research, Asia, Oceania, Gross national product, Gross domestic product, indexed journal

## Abstract

By and large, biomedical research is not a priority sector in Asian countries due to many factors. Lack of resources and trained manpower are certainly among these factors. We investigated the factors related to biomedical research productivity in Asian countries based on Medline data. The number of biomedical articles published in the indexed journals from each country of Asia and Oceania during 1990-1998 was used as a surrogate of total biomedical research productivity. Multiple regression analysis revealed that low gross national product per capita (p<0.013), insufficient number of physicians (p<0.047), and inadequate public spending on the health sector (p<0.049) were responsible for the meager number of biomedical publications in Asian countries.

## INTRODUCTION

The reports on the number of biomedical publications of 20 top-ranking countries in the world, European union countries, and Asian countries have already been published^1^^-^^4^^)^. It is a harsh reality that most of the developing countries have neither adequate resources nor trained personnel for conducting sound biomedical research. Among Asian countries, Israel was ranked top on the list when the number of biomedical articles were normalized to per million population and per billion dollar (US$) gross domestic product (GDP) per year, while Singapore was on the top when it was normalized to number of physicians (Table 1)^4^^)^. However, factors related to volume of biomedical publications are not examined yet. We considered Medline database as the reflection of standard research activities in each country as it is very difficult to quantify it by other means. The objective of this study is to elicit factors associated with low biomedical research productivity in Asian countries with a view to suggest macro level change to improve it. There are wide variations in gross national product (GNP) per capita in Asian countries. Asia is probably the only continent where low-income, middle-income and high-income countries are well-represented. Therefore, we included this region to examine the factors related to biomedical research productivity.

**Table 1.** Top ten countries in Asia and Oceania region based on publications per million population, per 1000 physicians, and per 1 billion US$ GDP.

Number of publications per million population per year		Number of publications per 1,000 physicians per year		Number of publications per I billion US$ GDP per year	
Israel	587	Singapore	244	Israel	35.9
Australia	304	Israel	128	New Zealand	15.3
New Zealand	246	New Zealand	117	Australia	14.7
Singapore	211	Japan	111	Lebanon	12.2
Japan	196	Australia	103	Papua New Guinea	10.6
Hong Kong	114	Papua New Guinea	55	Jordan	9.6
Taiwan	95	Thailand	32	Mongolia	8.8
Kuwait	53	Kuwait	30	India	6.8
Lebanon	45	Lebanon	24	Singapore	6.6
Saudi Arabia	23	Malaysia	20	Japan	5.9

## MATERIALS AND METHODS

Medline database was searched in October, 1999 to obtain research volume in Asian countries (including Oceania) with the use of the internet provider PubMed, according to the method described by Thompson^5^^)^. First, advanced-search option was selected and, then “publication date” (1990-1998) was entered to find out the total number of publications published during 1990-1998. After that, “affiliation” field was searched for each country of Asia and Oceania. Data obtained by this process were normalized to publication per million population per year for each of the countries. We selected 1990 as the first year for the search, because reliable data on authors’ affiliations were not available through PubMed before 1990. The data on population, GNP per capita, public expenditure on the health sector (% of GDP), number of physicians, and the number of indexed journal (s) published in each country were obtained from the reports published by World Bank (http://www.worldbank.org/data/countrydata/countrydata.html), United Nation Development Programme (http://www.undp.org/hdro/health.htm), and National Library of Medicine (http://www.nlm.nih.gov/tsd/serials/lji.html). Multiple regression analysis was performed to elicit significant factors related to biomedical research productivity. Publications per million population per year was considered as dependent variable in logarithm scale. The independent variables were GNP per capita, public expenditure on the health sector (% of GDP), physician size per thousand population, and the number of indexed journal (s) published from each country. Besides, Spearman rank correlation coefficient procedure was also performed to see the relationship of each of the independent variables with the dependent variable. All data were analyzed using STATA statistical software^6^^)^.

## RESULTS

**Figure 1.**
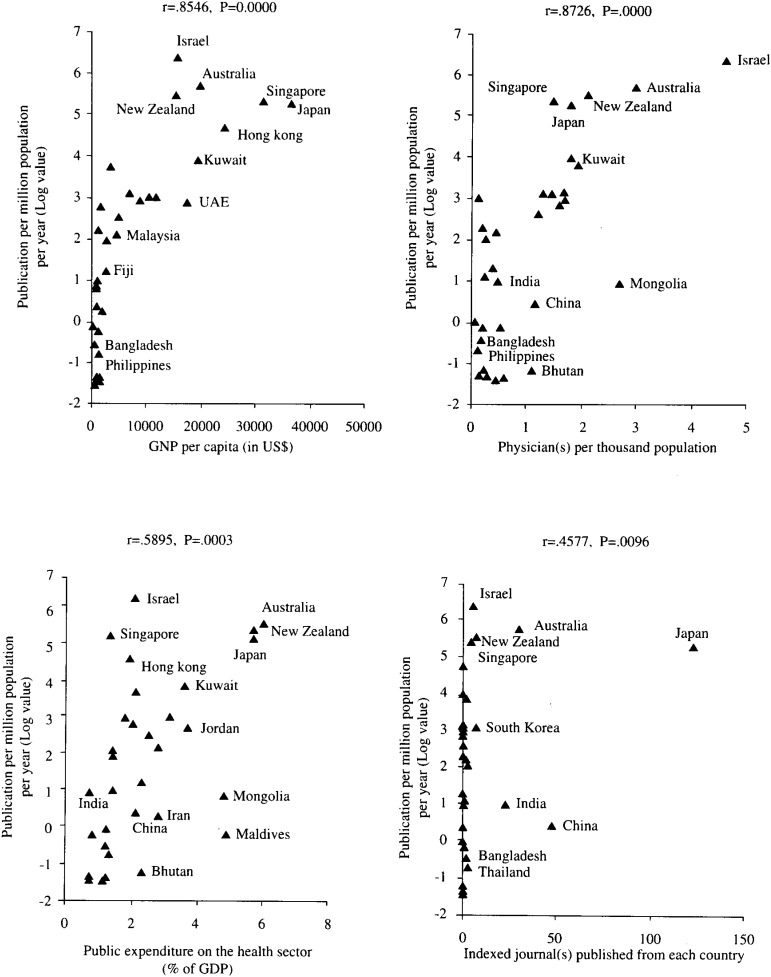
Relationship of publications per million populations per year (with Spearman’s rank correlation coefficient and p values) with GNP per capita (in US$), physician(s) per thousand population, public expenditure on the health sector (% of GDP), and the number of indexed journal (s) published from each of the countries.

Information on all the variables was available regarding 31 Asian and Oceania countries. Publications per year were ranged from 0.2 to 24,735 (0.13 to 587.9 per million population per year). GNP per capita and the number of physicians per thousand population were US$ 220-38,160 and 0.05-4.59, respectively. Variations were also observed regarding the number of indexed journal(s) published from each of the countries (0 to 123) and public expenditure on the health sector (0.7 to 5.7%). Multiple regression analysis showed that GNP per capita (p<0.013), number of physicians per thousand population (p<0.047), and public expenditure on the health sector (% of GDP) (p<0.049) were significantly related to biomedical research productivity (r^2^ = 0.75) (Table 2). Relationship of publications per million population per year with GNP per capita, physician(s) per thousand population, public expenditure on the health sector (% of GDP), and number of indexed journal(s) published from each country are shown in Figure 1.

**Table 2.** Results of multiple regression.

Independent variables	Coefficient	Standard error	t value	P>t
Physician(s) per thousand population	0.0259741	0.0097499	2.66	0.013
GNP per capita	0.0000955	0.0000457	2.09	0.047
Public spending on the health sector (% of GDP)	0.3681413	0.1780148	2.07	0.049
Number of indexed joumal(s) published in each country	-0.0180578	0.0127147	-1.42	0.167

R-squared value = 0.75

Dependent variable = Biomedical publications per million population per year (in logarithm scale)

## DISCUSSION

The number of publications elicited from Medline is only a gross estimate of research productivity irrespective of their quality. Besides, many local journals are being published in each country and many of those are not cited in Medline. In particular, in Japan and China, many journals are being published in their respective languages and most of them are not included in Medline database. On the other hand, in many countries, journals are being published in English but not included in Medline database, although most of these Journals are recognized by respective country’s research council. Besides, possibly in every country, researches are also conducted at non-governmental and international organizations and then remain as intramural reports rather than to be published in peer-reviewed journals. So, if these were included, actual number of research output could be much higher than the current results. There may be other factors related to biomedical research productivity. For example, health sector’s expenditures on the research and development sector, availability of trained manpower, facilities for research manpower development, government policy, and general attitude towards research, might have some linkage with research output. However, no systematic data exist on these factors at the present time. It is therefore worthwhile to have a gross impression about the probable factors related to biomedical research productivity.

In conclusion, low GNP per capita, insufficient number of physicians, and inadequate public spending on the health sector are related to the meager number of biomedical publications in Asian countries.
